# Machine Learning Models to Predict Metabolic Dysfunction‐Associated Steatotic Liver Disease (MASLD) With Simple Anthropometric and Biochemical Variables: A Cross‐Sectional Study in US Population

**DOI:** 10.1155/ijh/8221645

**Published:** 2026-04-01

**Authors:** Shiying Du, Hailiang Yu, Jianbo Du, Yunan Xu

**Affiliations:** ^1^ Comprehensive Supervision and Service Center of Hebei Health Commission, Shijiazhuang, China; ^2^ Department of Medicine, Beijing Kawin Technology Share-Holding Co. Ltd, Beijing, China; ^3^ Department of Medical Research, The First Affiliated Hospital of Guangxi Medical University, Nanning, China, gxmu.edu.cn

**Keywords:** machine learning, metabolic dysfunction-associated steatotic liver disease, National Health and Nutrition Examination Survey

## Abstract

**Background:**

Metabolic dysfunction‐associated steatotic liver disease (MASLD) is an emerging global health concern. This study was aimed at exploring the feasibility of utilizing machine learning (ML) algorithms to predict MASLD in large general populations based on simple anthropometric and biochemical parameters.

**Methods:**

Data from the 2017–2020 cycles of the US National Health and Nutrition Examination Survey (NHANES) were utilized. A total of 6814 participants (53.0% female) with complete transient elastography data were included. MASLD was defined as a controlled attenuation parameter ≥ 280 dB/m, with cardiometabolic risk factor and without excessive alcohol use. Key characteristics and biomarkers associated with MASLD were identified using the least absolute shrinkage and selection operator (LASSO) and the Boruta algorithms. ML methods, including logistic regression (LR), extreme gradient boosting (XGBoost), bootstrap aggregating, random forest, naive Bayes, light gradient boosting machine (LightGBM), decision tree, and support vector machines, were employed to develop the MASLD prediction models.

**Results:**

The median age of the 6814 participants was 53 years (interquartile range: 37~65). MASLD was detected among 2611 (38.3%) participants. Key predictors selected via LASSO and Boruta algorithms included body weight, standing height, waist circumference, diagnosis of diabetes, alanine aminotransferase, aspartate aminotransferase, and gamma glutamyl transferase. The areas under the receiver operating characteristic curves of LR, XGBoost, and other ML models were 0.841, 0.837, 0.815, 0.838, 0.814, 0.842, 0.796, and 0.828 in the internal validation cohort. Results indicate that LR, XGBoost, and LightGBM models outperform other models in predicting MASLD.

**Conclusions:**

The ML models of LR, XGBoost, and LightGBM are effective and simplified tools for predicting MASLD in the US general population. This study underscores the potential of ML models with simple noninvasive biomarkers in enhancing early detection and personalized management of fatty liver disease.

## 1. Background

Metabolic dysfunction‐associated steatotic liver disease (MASLD), previously termed metabolic dysfunction‐associated fatty liver disease (MAFLD) or nonalcoholic fatty liver disease (NAFLD), has seen a rapid global increase, overtaking viral hepatitis to become the most prevalent chronic liver disorder [1–4]. Studies have shown that MASLD can lead to a variety of more severe conditions, such as advanced fibrosis or cirrhosis [3], metabolic and cardiovascular diseases [5], hepatocellular carcinoma (HCC), or even mortality [5–7]. Early detection of MASLD is therefore critical, facilitating the implementation of appropriate medical and lifestyle interventions.

The liver biopsy was considered the definitive standard for diagnosing MASLD; however, its invasive nature, high cost, and potential procedure‐related complications limit its routine use [8]. Consequently, noninvasive markers, including blood tests and imaging techniques, have been increasingly applied to assess fatty liver disease [9, 10]. For example, higher alanine aminotransferase (ALT)/aspartate aminotransferase (AST) ratio may be independently associated with a significantly higher risk of NAFLD among American cohorts [11]. There are studies showing the association of a higher controlled attenuation parameter (CAP), which indicates the presence of hepatic steatosis, with increased age, body mass index (BMI), waist‐to‐hip ratio, diabetes, hypertension, ALT, and serum uric acid‐to‐high‐density lipoprotein cholesterol ratio [12, 13]. Triglyceride glucose (TyG), TyG‐BMI, and TyG‐waist circumference indices have been developed and validated to predict both the prevalence and mortality of MASLD [14–16]. Despite these advances, a simple and effective noninvasive screening tool for MASLD remains limited.

Recent advances in machine learning (ML) have significantly expanded the possibilities for predicting the risk of MASLD using demographic, clinical, and biochemical factors [4, 17]. ML algorithms, such as logistic regression (LR), random forest (RF), and extreme gradient boosting (XGBoost), have demonstrated potential in modeling complex, nonlinear relationships between predictors and outcomes [18–20]. These models can incorporate a broad spectrum of variables and achieve higher prediction accuracy compared to traditional statistical methods. However, many existing predictive models were established for specific populations [21] or fibrosis among MASLD patients, which restricts their generalizability across broader contexts [22, 23]. There are few MASLD prediction ML models tailored to general populations [20, 24, 25]. Also, some ML models for the general population included too many predictors or without liver‐related biomarkers, affecting the wide use or accuracy [20, 26]. Even though the liver ultrasound CAP measures could be used for the noninvasive diagnosis of MASLD, the availability and usage of liver ultrasound CAP are restrained by the geography or cost. Developing a predictive model for MASLD applicable to the general population with easily available predictors could facilitate early detection and timely intervention in the general population.

In this study, we aim to develop feasible and simplified MASLD prediction models using data from the 2017–2020 cycles of the National Health and Nutrition Examination Survey (NHANES) dataset, representative sample of the US general population. We compare the performance of various ML models and evaluate their ability to predict MASLD. Our findings enable to provide valuable insights into the clinical utility of a MASLD prediction framework among general populations, particularly in resource‐limited primary healthcare settings.

## 2. Methods

### 2.1. Study Design and Populations

This cross‐sectional study used data obtained from the 2017–2020 NHANES survey, which includes a wide range of clinical, demographic, and biochemical data [4, 12, 27]. All participants of the NHANES survey have provided written informed consent. The analysis protocol for this study was approved by the Ethics Committee of the First Affiliated Hospital of the Guangxi Medical University (IRB NO.: 2023‐K61‐01). Participants were not involved in the design, conduct, reporting, or dissemination plans of this research. The inclusion criteria were with complete liver elastography exam (i.e., fasting time of at least 3 h, 10 or more complete stiffness [E] measures, a liver stiffness interquartile [IQRe] range/median E < 30%, and available CAP measures). The exclusion criterium was aged < 20 years or excessive alcohol use (more than four drinks per day) [4, 12].

### 2.2. Data Collection and Measure

Candidate variables included 32 predictors including demographic variables (age, gender, and education level), physical assessment (body weight, standing height, waist circumference, and hip circumference), disease history (hypertension, diabetes, and kidney diseases) or lifestyle behaviors (smoking and alcohol consumption), and laboratory values (ALT, AST, alkaline phosphatase [ALP], albumin, blood urea nitrogen [BUN], creatinine, glucose, GGT, lactate dehydrogenase [LDH], total bilirubin, white blood cell count [WBC], hemoglobin, lymphocyte number, segmented neutrophils, platelet count [PLT], direct high‐density lipoprotein [HDL]‐cholesterol, low‐density lipoprotein [LDL]‐cholesterol, triglyceride, creatine phosphokinase [CPK], and total cholesterol) from 2017–2020 NHANES. The BMI is not included due to the collinearity with weight and height which are considered the predictors.

### 2.3. Diagnosis of MASLD

The primary outcome in this study was MASLD, defined as (1) CAP ≥ 280 dB/m among the participants with complete CAP exams by FibroScan (Echosens, France) in the NHANES transient elastography dataset; (2) present with at least one of five cardiometabolic risk factors: (a) BMI ≥ 25 kg/m^2^, (b) diagnosed with Type 2 diabetes (DIQ010—Doctor told you have diabetes from the questionnaire data of NHANES), (c) high blood pressure (BPQ020—Ever told you had high blood pressure from the questionnaire data of NHANES), (d) plasma triglycerides ≥ 1.70 mmol/L, or (e) plasma HDL‐cholesterol ≤ 1.0 mmol/L; and (3) without excessive alcohol use, defined as having > 4 alcoholic drinks (One drink means a 12‐oz. beer, a 5‐oz. glass of wine, or one and a half ounces of liquor.) [2, 28, 29].

### 2.4. Data Preprocessing and Feature Selection

For the continuous variables, the missing values were imputed with median values, and all variables were standardized to be included in models. Variables, including age (20–29, 30–49, 50–69, 70 years), gender (female or male), race (Hispanic, non‐Hispanic White, non‐Hispanic Black, or non‐Hispanic Asian/Other), education level (less than high school, high school graduate, or college or above), marital status (married/living with partner, widowed/divorced/separated, or never married/other), hypertension (yes or no), diabetes (yes or no), kidney diseases (yes or no), smoking (yes or no), and alcohol consumption (yes or no), were used as categorical variables. The missing value of these categorical variables was treated as a separate subgroup or combined with another subgroup.

For feature selection, we employed the least absolute shrinkage and selection operator (LASSO) regression and the Boruta algorithm. LASSO regression, which is effective in handling multicollinearity and enhancing model interpretability with L1 regularization, was applied to refine the variables by reducing the coefficients of less important predictors to zero. We utilized the glmnet package in R for LASSO regression of binary outcome data with 10‐fold cross‐validation. The Boruta algorithm, a robust and stable method built around RFs, was used to identify relevant variables by comparing the importance of each predictive variable. We used Boruta package in R to conduct this algorithm.

The overlapped variables by LASSO and Boruta algorithm were selected. Additionally, we set the number of predictable features into 10 to meet the demands of simplicity and feasibility at primary healthcare settings or self‐evaluation by general populations. If the selected variables were closely related to each other (such as diagnosis of diabetes and glucose), only the more easily available one was included based on medical expertise and expert suggestions, and the feature selection process was redone to ensure eight variables at most to be selected in the final model.

### 2.5. Development and Evaluation of ML Models

We applied the following ML models to predict MASLD with features selected by LASSO and Boruta algorithms: LR, XGBoost, bootstrap aggregating (Bagging), RF, naive Bayes (NB), light gradient boosting machine (LightGBM), decision tree (DT), and support vector machine (SVM) [30]. Each model was trained on 70% of the data, validated internally on another 20% of the data, and the remaining 10% was used for internal testing [20]. LR is a basic method for binary classification that estimates the probability of a given categorical outcome based on a set of predictor variables. In the LR model, glm function in Base R was used to get the results with classical LRs.

XGBoost is a gradient boosting method known for its high performance in large datasets, building multiple DTs to improve prediction accuracy. We set the learning rate of 10% and the maximum depth of a tree to four to limit the model′s complexity and prevent overfitting.

To enhance the model′s stability and accuracy, bagging was implemented which generates multiple resampled training subsets and combines predictions from individual base models. In this study, we employed a bagging model based on DTs using the bagging () function of the “ipred” package in R. The model was trained with the parameters for the number of bootstrap replications of 25 and coob value of “true” to enable out‐of‐bag estimates, providing an unbiased estimation of model performance.

RF, an ensemble approach that utilizes DTs as base classifiers, was also applied. RF constructs multiple trees by recursively partitioning the data, and final predictions are determined by a voting mechanism. The “randomForest” package in R was used with the Gini Index as the split criterion, 500 trees, and no restriction on maximum tree depth.

NB, a probabilistic classifier derived from Bayes′ theorem under the assumption of feature independence, was implemented using the naiveBayes () function from the “e1071” package in R. This model is known for its robust performance with categorical and continuous data.

We further employed LightGBM, a gradient boosting DT framework optimized for efficiency and accuracy. The model was constructed using the lgb.train() function from “lightgbm” package in R to develop a binary classification model for predicting the risk of MASLD with the area under the curve (AUC) as the primary evaluation metric. The parameter of learning rate was set to 0.05, the complexity of individual trees to 31, and a total of boosting rounds to 100.

DT is a nonlinear model that splits the dataset into distinct groups based on specific threshold values of predictor variables. We employed a DT classifier using the rpart() function from the “rpart” package in R. The model was constructed using the classification method with the minimum split size set to 20 to get better generalization.

SVM, a supervised learning algorithm that identifies the optimal hyperplane to maximize the margin between class separation, was applied using the svmRadial method from the “caret” package in R with a Radial Basis Function kernel and five‐fold cross‐validation. This approach is particularly effective in high‐dimensional feature spaces and when class boundaries are nonlinear [20].

The performance of all ML models was evaluated using ROC curves, AUC with 95% CI, sensitivity, specificity, accuracy, precision, and F1‐score [20].

### 2.6. Model Interpretability With SHAP

To interpret model outputs, we used Shapley additive explanations (SHAP), which quantify the contribution of each feature to the prediction [30]. SHAP calculates the average marginal contribution of individual features on the model′s output by comparing all feasible feature combinations, allowing for both global and local interpretation of model behavior [31]. For each feature, SHAP values represent its mean impact on model predictions across all observations.

### 2.7. Statistical Analyses

Continuous variables were expressed as medians with interquartile ranges (IQRs) for non‐normally distributed data. Categorical variables were presented as counts and percentages. Comparisons between participants with and without MASLD were performed using the chi‐squared tests for categorical variables and Mann–Whitney *U* tests for continuous variables. A two‐sided *p* value < 0.05 was considered statistically significant. All analyses were conducted using SAS Version 9.4 (SAS Institute Inc., Cary, North Carolina, USA) and R Version 4.4.3 (R Foundation for Statistical Computing, Vienna, Austria).

## 3. Results

### 3.1. Baseline Characteristics

**FIGURE 1:**
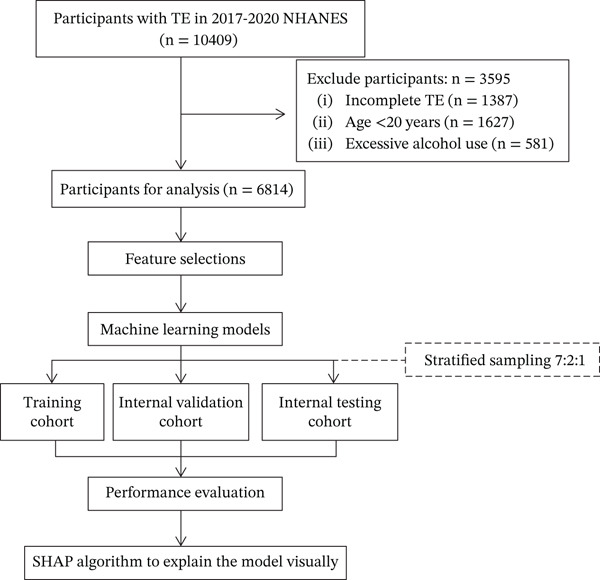
Flowchart of the STUDY. NHANES: National Health and Nutrition Examination Survey; SHAP: SHapley additive explanations; TE: transient elastography.

A total of 6814 participants of 2017–2020 NHANES were included in this study. The flowchart of the study was shown in Figure 1. The median age (IQR) was 53 (37~65) years, female accounting for 53.0%. Among the participants, 58.9% were with college level or above, and 58.7% were married or living with partners.

MASLD was detected among 2611 (38.3%) participants. Between MASLD and non‐MASLD participants, there were significant differences in age, gender, race, education level, marital status, history of hypertension, diabetes, and smoking (Table 1). The differences in height, weight, BMI, waist circumference, hip circumference, ALT, AST, ALP, albumin, BUN, glucose, GGT, LDH, total bilirubin, WBC, hemoglobin, PLT, triglyceride, CPK, and total cholesterol between MASLD and non‐MASLD groups were also statistically significant. Baseline characteristics of patients in the training, internal validation, and internal testing cohorts were shown in Table S1. In Table S1, the baseline characteristics, including frequency distributions of age, gender, race, education level, marital status, hypertension, diabetes, kidney disease, smoking, drinking, and MASLD, were presented by the groups of training, internal validation, and internal testing cohorts. The median and IQR of anthropometric and biochemical variables were also shown.

**TABLE 1:** Comparison of general characteristics of NHANES 2017–2020 cohorts.

Characteristics	Total *N* = 6814	Non‐MASLD *n* = 4203	MASLD *n* = 2611	*p* value
Age (years)	53 (37, 65)	50 (34, 64)	56 (42, 66)	< 0.001
20–29	950 (13.9%)	735 (17.5%)	215 (8.2%)	
30–49	2107 (30.9%)	1342 (31.9%)	765 (29.3%)	
50–69	2584 (37.9%)	1410 (33.5%)	1174 (45.0%)	
70–	1173 (17.2%)	716 (17.0%)	457 (17.5%)	
Gender				< 0.001
Male	3200 (47.0%)	1827 (43.5%)	1373 (52.6%)	
Female	3614 (53.0%)	2376 (56.5%)	1238 (47.4%)	
Race				< 0.001
Hispanic	1423 (20.9%)	769 (18.3%)	654 (25.0%)	
Non‐Hispanic White	1219 (17.9%)	791 (18.8%)	428 (16.4%)	
Non‐Hispanic Black	1862 (27.3%)	1273 (30.3%)	589 (22.6%)	
Non‐Hispanic Asian/Other	2310 (33.9%)	1370 (32.6%)	940 (36.0%)	
Education level				0.013
Less than high school	1598 (23.5%)	961 (22.9%)	637 (24.4%)	
High school graduate	1202 (17.6%)	709 (16.9%)	493 (18.9%)	
College or above	4014 (58.9%)	2533 (60.3%)	1481 (56.7%)	
Marital status				< 0.001
Married/living with partner	4001 (58.7%)	2351 (55.9%)	1650 (63.2%)	
Widowed/divorced/separated	1286 (18.9%)	904 (21.5%)	382 (14.6%)	
Never married/other	1527 (22.4%)	948 (22.6%)	579 (22.2%)	
Hypertension history				< 0.001
Yes	2625 (38.5%)	1329 (31.6%)	1296 (49.6%)	
No	4189 (61.5%)	2874 (68.4%)	1315 (50.4%)	
Diabetes history				< 0.001
Yes	1019 (15.0%)	384 (9.1%)	635 (24.3%)	
No	5795 (85.0%)	3819 (90.9%)	1976 (75.7%)	
Kidney disease history				0.282
Yes	259 (3.8%)	151 (3.6%)	108 (4.1%)	
No	4120 (60.5%)	2592 (61.7%)	1528 (58.5%)	
Smoking				0.011
Yes	2694 (39.5%)	1611 (38.3%)	1083 (41.5%)	
No	4120 (60.5%)	2592 (61.7%)	1528 (58.5%)	
Drinking > once a week				0.003
Yes	1595 (23.4%)	1034 (24.6%)	561 (21.5%)	
No	5219 (76.6%)	3169 (75.4%)	2050 (78.5%)	
Height (cm)	166.0 (159.0, 173.5)	165.6 (158.7, 173.1)	166.4 (159.6, 174.2)	< 0.001
Weight (kg)	79.2 (67.1, 93.9)	72.7 (62.1, 84.4)	90.8 (77.9, 106.3)	< 0.001
Body mass index (kg/m^2^)	28.6 (24.7, 33.2)	26.2 (23.2, 30.0)	32.2 (28.6, 37.2)	< 0.001
Waist circumference (cm)	98.9 (88.9, 109.7)	93.2 (83.9, 101.8)	108.3 (98.9, 119.2)	< 0.001
Hip circumference (cm)	104.4 (97.2, 113.5)	101.1 (94.6, 108.3)	110.1 (103.4, 121.3)	< 0.001
ALT (U/L)	18 (13, 24)	16 (12, 21)	20 (16, 30)	< 0.001
AST (U/L)	19 (16, 23)	19 (16, 22)	19 (16, 24)	< 0.001
ALP (IU/L)	74 (62, 88)	73 (60, 85)	76 (66, 92)	< 0.001
Albumin (g/L)	41 (39, 43)	41 (39, 43)	41 (38, 42)	< 0.001
Blood urea nitrogen (mmol/L)	5.00 (3.93, 6.07)	5.00 (3.93, 6.07)	5.00 (4.28, 6.07)	< 0.001
Creatinine (*μ*mol/L)	74.26 (62.76, 87.52)	74.26 (62.76, 87.52)	74.26 (62.76, 88.40)	0.318
Glucose (mmol/L)	5.16 (4.83, 5.66)	5.11 (4.77, 5.44)	5.38 (5.05, 6.22)	< 0.001
GGT (IU/L)	21 (15, 30)	19 (13, 26)	24 (19, 38)	< 0.001
LDH (IU/L)	154 (138, 173)	154 (137, 171)	154 (141, 174)	< 0.001
Total bilirubin (*μ*mol/L)	6.84 (5.13, 8.55)	6.84 (5.13, 8.55)	6.84 (5.13, 8.55)	0.035
WBC (1000 cells/*μ*L)	6.8 (5.6, 8.2)	6.7 (5.4, 7.9)	7.1 (6.1, 8.7)	< 0.001
Hemoglobin (g/dL)	14.1 (13.1, 14.9)	13.9 (13.0, 14.8)	14.2 (13.3, 15.2)	< 0.001
Lymphocyte number (1000 cells/*μ*L)	2.1 (1.7, 2.6)	2.1 (1.6, 2.5)	2.2 (1.8, 2.7)	< 0.001
Segmented neutrophils (1000 cell/*μ*L)	3.9 (3.0, 4.9)	3.8 (2.9, 4.7)	4.1 (3.3, 5.2)	< 0.001
Platelet count (1000 cells/*μ*L)	239 (204, 279)	239 (202, 277)	239 (207, 284)	< 0.001
Direct HDL‐cholesterol (mmol/L)	1.32 (1.11, 1.58)	1.40 (1.22, 1.68)	1.22 (1.03, 1.40)	< 0.001
Triglyceride (mmol/L)	2.741 (2.741, 2.741)	2.741 (2.741, 2.741)	2.741 (2.741, 2.741)	0.041
CPK (IU/L)	1.010 (1.010, 1.010)	1.010 (0.881, 1.010)	1.010 (1.010, 1.253)	< 0.001
Total cholesterol (mmol/L)	4.73 (4.14, 5.40)	4.73 (4.11, 5.33)	4.73 (4.14, 5.46)	0.002

*Note:* The categorical data were presented with frequencies and percentages. Continuous variables were presented with median (interquartile range). *p* values were generated with chi‐squared tests and nonparametric tests for categorical and continuous variable, respectively.

Abbreviations: ALP, alkaline phosphatase; ALT, alanine aminotransferase; AST, aspartate aminotransferase; CPK, creatine phosphokinase; GGT, gamma glutamyl transferase; HDL, high‐density lipoprotein; LDH, lactate dehydrogenase; MASLD, metabolic dysfunction‐associated steatotic liver disease; WBC, white blood cell count.

### 3.2. Feature Selection

The predictable variables selected by LASSO and Boruta algorithms, after adjustment with clinical expert suggestions, were body weight, standing height, waist circumference, ALT, AST, GGT, and diagnosis of diabetes (Have any doctor told you have diabetes?). (Figure S1 showed the features selected by LASSO and Boruta algorithms. In Figure S1, both methods identified weight, height, waist circumference, ALT, AST, GGT, and diagnosis of diabetes [DB] as predictors. In addition, LASSO algorithm exclusively selected PLT and BUN, while Boruta algorithm uniquely identified hip circumference.)

### 3.3. Assessment of the Accuracy of ML Models for the Diagnosis of MASLD

The performances of all the ML models were evaluated with AUC, accuracy, sensitivity, specificity, precision, and F1‐score by internal validation cohort and internal testing cohort (Table 2). The LR, XGBoost, RF, and LightGBM models achieved higher AUC (0.841, 0.837, 0.838, and 0.842) than other models in the internal validation cohort, indicating strong predictive ability for MASLD with the selected features. In the internal testing cohort, the LR, XGBoost, RF, and LightGBM models achieved higher AUC (0.817, 0.815, 0.813, and 0.825) than other models. The Bagging, NB, DT, and SVM models also achieved AUCs > 0.75 (Figure 2).

**TABLE 2:** Performance of machine learning models for predicting MASLD in NHANES 2017–2020 cohorts.

Cohort	Model	AUC	95%CI AUC	Optimal cutoff	Accuracy	Sensitivity	Specificity	Precision	F1‐score
Internal validation cohort	LR	0.841	0.820~0.861	0.373	0.767	0.761	0.771	0.674	0.715
	XGBoost	0.837	0.816~0.858	0.389	0.761	0.785	0.745	0.657	0.716
	Bagging	0.815	0.792~0.837	0.420	0.742	0.728	0.751	0.645	0.684
	RF	0.838	0.817~0.859	0.327	0.750	0.866	0.679	0.626	0.727
	NB	0.814	0.792~0.837	0.181	0.747	0.751	0.745	0.647	0.695
	LightGBM	0.842	0.822~0.863	0.392	0.767	0.785	0.756	0.667	0.721
	DT	0.796	0.773~0.820	0.355	0.740	0.782	0.714	0.630	0.697
	SVM	0.828	0.805~0.850	0.380	0.773	0.716	0.808	0.699	0.708
Internal testing cohort	LR	0.817	0.786~0.848	0.366	0.743	0.709	0.764	0.651	0.679
	XGBoost	0.815	0.784~0.847	0.430	0.756	0.732	0.771	0.666	0.697
	Bagging	0.797	0.764~0.830	0.300	0.705	0.835	0.624	0.580	0.684
	RF	0.813	0.781~0.844	0.305	0.711	0.839	0.631	0.586	0.690
	NB	0.786	0.752~0.820	0.192	0.724	0.686	0.748	0.628	0.656
	LightGBM	0.825	0.794~0.855	0.313	0.722	0.858	0.638	0.596	0.703
	DT	0.768	0.732~0.804	0.355	0.714	0.747	0.693	0.602	0.667
	SVM	0.799	0.764~0.833	0.346	0.756	0.701	0.790	0.675	0.688

Abbreviations: AUC, area under the curve; Bagging, bootstrap aggregating; DT, decision tree; LightGBM, light gradient boosting machine; LR, logistic regression; MASLD, metabolic dysfunction‐associated steatotic liver disease; NB, naive Bayes; NHANES, National Health and Nutrition Examination Survey; RF, random forest; SVM, support vector machines; XGBoost, extreme gradient boosting.

FIGURE 2Prediction performance by AUCs of (a) internal validation and (b) internal testing cohorts for the machine learning models for predicting MASLD in NHANES 2017–2020 cohorts. Abbreviations: AUC, area under the curve; Bagging, bootstrap aggregating; DT, decision tree; LightGBM, light gradient boosting machine; LR, logistic regression; MASLD, metabolic dysfunction‐associated steatotic liver disease; NB, naive Bayes; NHANES, National Health and Nutrition Examination Survey; RF, random forest; ROC, receiver operating characteristic; SVM, support vector machines; XGBoost, extreme gradient boosting.

**Figure:**
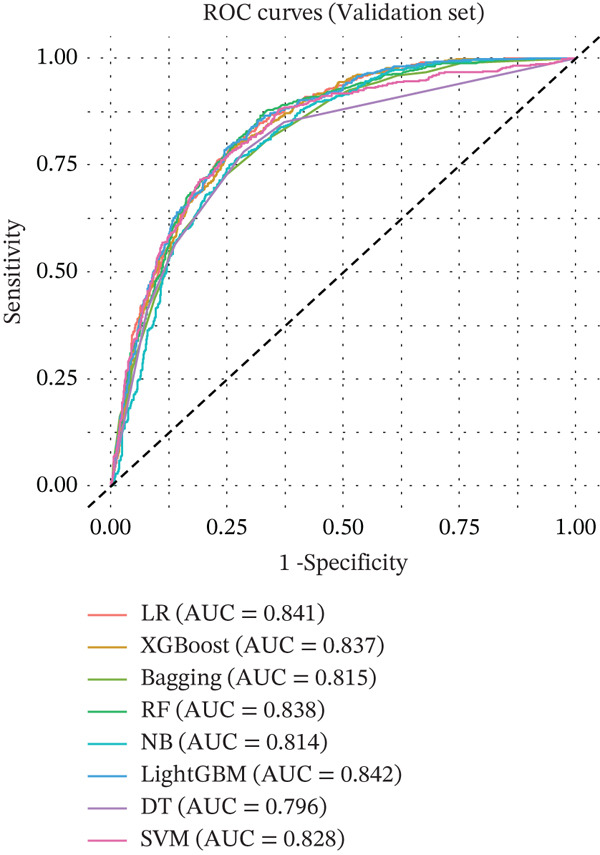
(a)

**Figure:**
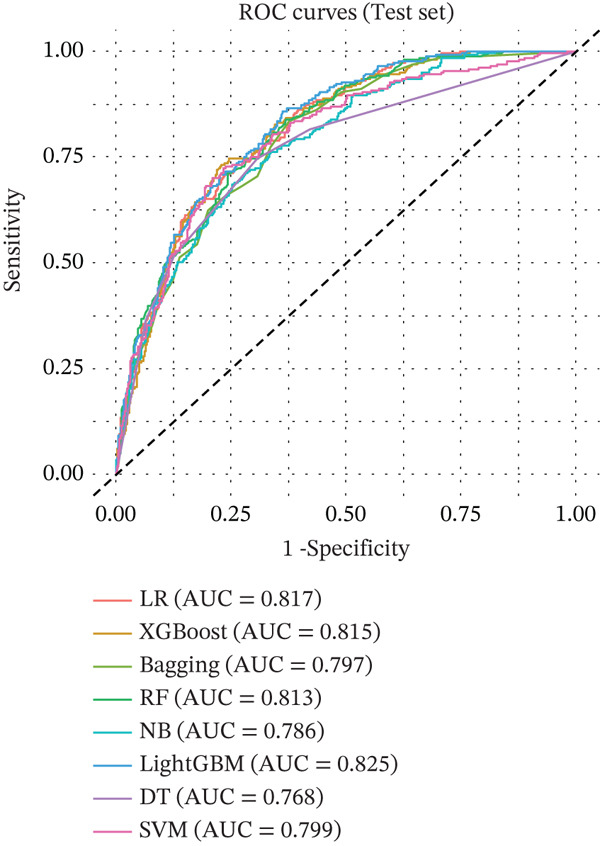
(b)

### 3.4. Visualization by SHAP

Figure 3a presents the feature importance ranking for the XGBoost model derived from SHAP values. Predictors were ordered by the mean absolute SHAP value, reflecting their relative contribution to the model′s output. The SHAP summary plot (Figure 3b) further depicts the direction and magnitude of each feature′s effect on model predictions. Higher SHAP values for specific predictors indicated an elevated risk of MASLD. Waist circumference, ALT, weight, height, and GGT emerged as the five most influential variables. For instance, patients exhibiting aberrant waist circumference were found to be predisposed to a higher incidence of MASLD, compared with those with normal waist circumference. Noninvasive liver‐related biochemical markers, such as ALT, AST, and GGT, also proved to be significant predictors of MASLD.

FIGURE 3(a) SHAP summary plot showing the distribution of the SHAP values of each feature; (b) SHAP feature importance shown according to the mean absolute SHAP value of each feature in NHANES 2017–2020 cohorts.

**Figure:**
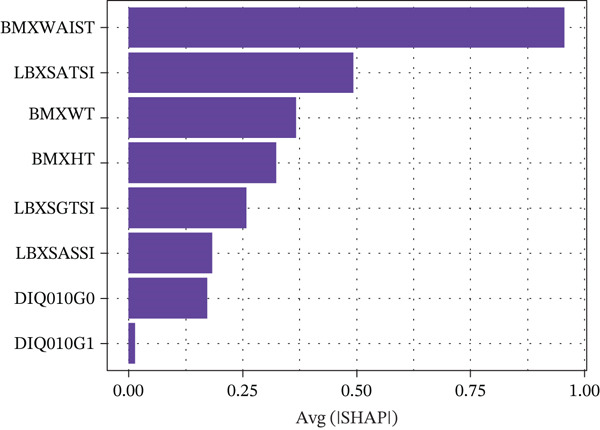
(a)

**Figure:**
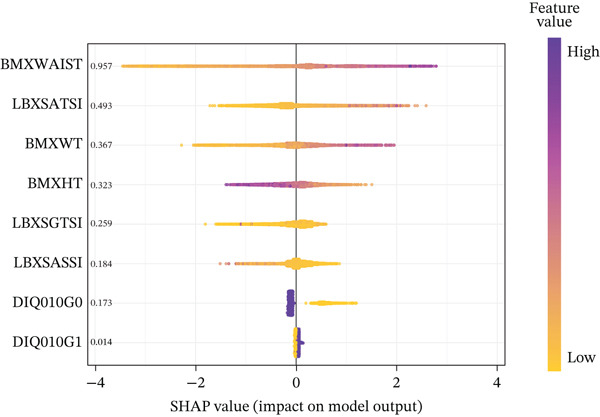
(b)

## 4. Discussion

This study demonstrated the potential of various ML models in predicting MASLD using commonly available anthropometric and biochemical variables via NHANES 2017–2020. The selected seven common features, including body weight, height, waist circumference, diabetes history, ALT, AST, and GGT, showed significant accuracy of the ML prediction models and high potential in wide use at resource‐limited primary healthcare setting or self‐evaluation scenarios.

The predictive power of ML methods highlights the benefits of incorporating anthropometric, clinical, and biochemical variables. The ML models by similar studies [32, 33] showed better efficacy than the traditional noninvasive indices such as FIB‐4, APRI, and Forns. Noninvasive biomarkers, such as weight, waist circumference, and diabetes, proved to be significant predictors of MASLD. These findings align with previous research that has highlighted the role of metabolic factors in MASLD development [2, 12, 19, 34]. The inclusion of liver‐related biomarkers, such as ALT, AST, and GGT, further enhances the ability to predict MASLD, as these biochemical variables are commonly available and reflect liver dysfunction and systemic inflammation [33]. In this study, similarly in other published results, the waist circumference is crucial to predict MASLD [32, 35].

Additionally, this study did not use other advanced biochemical markers, such as Apolipoprotein A‐1, hyaluronic acid, or tissue inhibitor of metalloproteinase 1, which may hinder the wide use of the prediction model in primary healthcare setting [10]. So, the ML models from this study possess high potential for wide use in resource‐limited settings. In resource‐limited settings where VCTE or TE is impractical, the ML model developed in our study may function as a low‐cost screening tool for MASLD. Only high‐risk individuals identified by the algorithm are referred for confirmatory VCTE or MRI‐PDFF tests, thus optimizing the use of limited imaging capacity and cutting down on patient expenses. The diagnosis of diabetes can also be represented by glucose‐related indicators such as fasting blood glucose or impaired glucose tolerance. However, self‐reported diabetes history or diagnosis from medical records is more convenient and widely available. Therefore, in the broader application of ML models, this predictor can be flexibly handled [2].

To our knowledge, this study is among the early studies to utilize ML models with feasible and simple anthropometric and biochemical predictors to predict the risk of MASLD in the general population of the United States. The models of LR, XGBoost, and LightGBM with simple predictors of body weight, standing height, waist circumference, ALT, AST, GGT, and diagnosis with diabetes outperform other models. There were some limitations for the study. First, the diagnosis of MASLD in our study was mainly based on FibroScan measurements and excluded the participants with excessive alcohol use, which may introduce certain errors due to factors like optimal cutoff values, operator variability, and patient conditions, and does not reflect the full metabolic features of MASLD, such as glucose and lipid abnormalities. Moreover, we did not use different criteria for HDL‐cholesterol or alcohol use. Second, the models have not been validated using external datasets. Therefore, its generalizability to other regions and populations may be confined. Future studies should incorporate external various databases, such as the NHANES 2021–2023 dataset, with some biochemical variables not available and requiring imputation, real‐world data from hospitals, or physical examination centers. Third, the study′s cross‐sectional design limits the ability to infer causal relationships or predict future risk of MASLD. However, the samples size and data quality from NHANES can guarantee the robustness and stability of the results [27].

## 5. Conclusion

Our study demonstrated that the ML models with simple anthropometric and biochemical measurements effectively identify MASLD in general populations. These findings provide valuable insights for clinical applications, as they can aid in early detection, less invasive screening, and personalized treatment and intervention planning for patients at risk of MASLD in a large general population. Future research should focus on further validating these models with external datasets, incorporating additional common biomarkers.

## Author Contributions

Y.X. and H.Y. conceived of and designed the study. S.D., J.D., and H.Y. performed the data curation and analysis and contributed to interpreting the results. Y.X. verified the analytical methods and results. S.D. drafted the manuscript. Y.X., H.Y., and J.D. revised the manuscript. Y.X. acts as a guarantor responsible for the overall content. S.D. and H.Y. have contributed equally.

## Funding

This work was supported by the Youth Natural Science Foundation of Guangxi (2024GXNSFBA010067 to Y.X.), the Clinical Research “Climbing” Program of the First Affiliated Hospital of Guangxi Medical University (YYZS2023015 to Y.X.), and the National Natural Science Foundation of China (82404382 to Y.X.).

## Disclosure

All authors have discussed the results, read, and approved the final manuscript. The funders had no role in study design, data collection, analysis, interpretation, or writing of the manuscript.

## Ethics Statement

The analysis protocol for this study was approved by the Ethics Committee of the First Affiliated Hospital of Guangxi Medical University (IRB NO.: 2023‐K61‐01).

## Conflicts of Interest

The authors declare no conflicts of interest.

## Supporting information


**Supporting Information** Additional supporting information can be found online in the Supporting Information section. Figure S1 Feature selection results using LASSO and Boruta algorithms. Table S1: Baseline characteristics and outcomes of patients in the training, internal validation, and internal testing cohorts in 2017–2020 NHANES.

## Data Availability

The NHANES data are publicly available at https://wwwn.cdc.gov/nchs/nhanes/search/datapage.aspx?Component=Examination%26Cycle=2017-2020.
