# Supplementary material for: Machine Learning Models to Predict Metabolic Dysfunction‐Associated Steatotic Liver Disease (MASLD) With Simple Anthropometric and Biochemical Variables: A Cross‐Sectional Study in US Population
Source: Int J Hepatol. 2026 Apr 1;2026:8221645. doi: 10.1155/ijh/8221645 (PMC13042330; doi:10.1155/ijh/8221645)
Supplement: Supplementary file 1 — Supporting Information Additional supporting information can be found online in the Supporting Information section. Figure S1 Feature selection results using LASSO and Boruta algorithms. Table S1: Baseline characteristics and outcomes of patients in the training, internal validation, and internal testing cohorts in 2017–2020 NHANES. [file IJH-2026-8221645-s001.docx]

**Supplementary materials:**

**Title:** Machine learning models to predict metabolic dysfunction-associated steatotic liver disease (MASLD) with simple anthropometric and biochemical variables: a cross-sectional study in United States population

- **Table S1** Baseline characteristics and outcomes of patients in the training, internal validation and internal testing cohort in 2017-2020 NHANES
- **Figure S1** Features selected by LASSO and Boruta algorithms

In **Table S1,** the baseline characteristics, including frequency distributions of age, gender, race, education level, marital status, hypertension, diabetes, kidney disease, smoking, drinking, and MASLD were presented by the groups of training, internal validation and internal testing cohorts. The median and interquartile range of anthropometric and biochemical variables were also showed.

**Figure S1** showed the features selected by LASSO and Boruta algorithms. In Figure S1, both methods identified weight, height, waist circumference, GGT, and diagnosis of diabetes (DB) as predictors. In addition, LASSO algorithm exclusively selected Age, AST, PLT and blood urea nitrogen (BUN) while Boruta algorithm uniquely identified ALT and hip circumference)


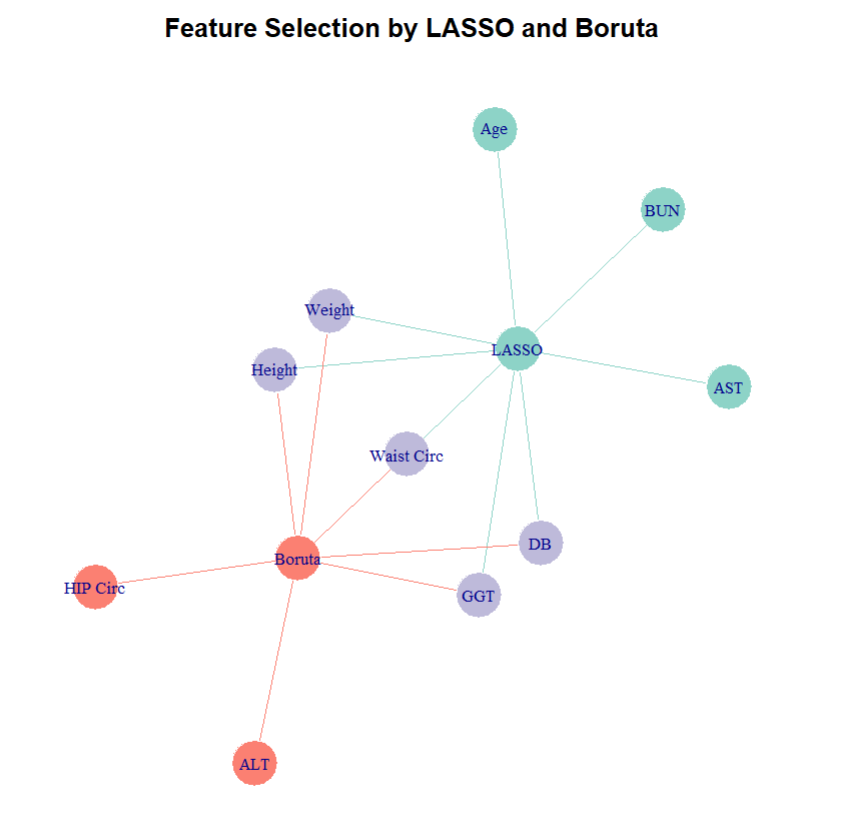


**Figure S1** Features selected by LASSO and Boruta algorithms

Abbreviations: ALT, alanine aminotransferase; AST, aspartate aminotransferase; Bun, blood urea nitrogen; DB: Diabetes history; GGT, gamma glutamyl transferase; Circ, circumference; LASSO: least absolute shrinkage and selection operator; PLT, Platelet count.

**Table S1** Baseline characteristics and outcomes of patients in the training, internal validation and internal testing cohort in 2017-2020 NHANES

|  | **Train cohort^1^** | **Internal validation cohort^1^** | **Internal testing cohort^1^** |  |
| --- | --- | --- | --- | --- |
| Characteristics | n = 4,771 | n = 1,362 | n = 681 | *P value*^2^ |
| Age Media (*IQR*) | 53 (37, 65) | 52 (37, 65) | 54 (37, 65) | 0.6 |
| 20-29 yrs | 648 (14%) | 200 (15%) | 102 (15%) |  |
| 30-49 yrs | 1,492 (31%) | 420 (31%) | 195 (29%) |  |
| 50-70 yrs | 1,824 (38%) | 505 (37%) | 255 (37%) |  |
| 70- yrs | 807 (17%) | 237 (17%) | 129 (19%) |  |
| Gender |  |  |  | 0.3 |
| Male | 2,210 (46%) | 663 (49%) | 327 (48%) |  |
| Female | 2,561 (54%) | 699 (51%) | 354 (52%) |  |
| Race |  |  |  | 0.6 |
| Hispanic | 981 (21%) | 284 (21%) | 158 (23%) |  |
| Non-Hispanic White | 866 (18%) | 247 (18%) | 106 (16%) |  |
| Non-Hispanic Black | 1,299 (27%) | 379 (28%) | 184 (27%) |  |
| Non-Hispanic Asian/Other | 1,625 (34%) | 452 (33%) | 233 (34%) |  |
| Education level |  |  |  | 0.9 |
| 1.Less than high school | 1,129 (24%) | 306 (22%) | 163 (24%) |  |
| 2.High school graduate | 839 (18%) | 248 (18%) | 115 (17%) |  |
| 3.College or above | 2,803 (59%) | 808 (59%) | 403 (59%) |  |
| Marital status |  |  |  | 0.074 |
| Married/Living with Partner | 2,828 (59%) | 758 (56%) | 415 (61%) |  |
| Widowed/Divorced/Separated | 876 (18%) | 281 (21%) | 129 (19%) |  |
| Never married/Other | 1,067 (22%) | 323 (24%) | 137 (20%) |  |
| Hypertension history |  |  |  | 0.2 |
| No | 2,925 (61%) | 861 (63%) | 403 (59%) |  |
| Yes | 1,846 (39%) | 501 (37%) | 278 (41%) |  |
| Diabetes history |  |  |  | 0.4 |
| No | 4,074 (85%) | 1,151 (85%) | 570 (84%) |  |
| Yes | 697 (15%) | 211 (15%) | 111 (16%) |  |
| Kidney disease history |  |  |  | 0.8 |
| No | 4,588 (96%) | 1,309 (96%) | 658 (97%) |  |
| Yes | 183 (3.8%) | 53 (3.9%) | 23 (3.4%) |  |
| Smoking |  |  |  | 0.7 |
| No | 2,879 (60%) | 819 (60%) | 422 (62%) |  |
| Yes | 1,892 (40%) | 543 (40%) | 259 (38%) |  |
| Drinking >once a week |  |  |  | 0.8 |
| No | 3,665 (77%) | 1,035 (76%) | 519 (76%) |  |
| Yes | 1,106 (23%) | 327 (24%) | 162 (24%) |  |
| Height (cm) | 166 (159, 173) | 166 (159, 174) | 166 (159, 174) | 0.7 |
| Weight (kg) | 79 (67, 94) | 78 (66, 94) | 79 (68, 93) | 0.7 |
| Body Mass Index (kg/m**2) | 29 (25, 33) | 28 (25, 33) | 29 (25, 33) | 0.5 |
| Waist Circumference (cm) | 99 (89, 110) | 99 (88, 110) | 99 (90, 109) | 0.5 |
| Hip Circumference (cm) | 104 (97, 114) | 104 (97, 113) | 104 (97, 112) | 0.4 |
| ALT (U/L) | 18 (13, 25) | 18 (13, 24) | 18 (13, 24) | 0.7 |
| AST (U/L) | 19 (16, 23) | 19 (16, 23) | 19 (16, 23) | 0.5 |
| ALP (IU/L) | 74 (62, 88) | 74 (61, 87) | 74 (63, 89) | 0.4 |
| Albumin (g/L) | 41.0 (39.0, 43.0) | 41.0 (39.0, 43.0) | 41.0 (39.0, 43.0) | 0.2 |
| Blood Urea Nitrogen (mmol/L) | 5.00 (3.93, 6.07) | 5.00 (4.28, 6.07) | 5.00 (4.28, 6.07) | >0.9 |
| Creatinine (umol/L) | 74 (63, 88) | 74 (64, 88) | 74 (64, 88) | 0.5 |
| Glucose (mmol/L) | 5.16 (4.83, 5.61) | 5.16 (4.83, 5.66) | 5.16 (4.83, 5.66) | 0.3 |
| GGT (IU/L) | 21 (15, 30) | 21 (15, 30) | 21 (14, 29) | 0.5 |
| LDH (IU/L) | 154 (138, 173) | 154 (137, 172) | 154 (140, 170) | 0.7 |
| Total Bilirubin (umol/L) | 6.8 (5.1, 8.6) | 6.8 (5.1, 8.6) | 6.8 (5.1, 8.6) | 0.6 |
| WBC (1000 cells/uL) | 6.80 (5.60, 8.20) | 6.80 (5.70, 8.20) | 6.80 (5.50, 8.20) | 0.6 |
| Hemoglobin (g/dL) | 14.10 (13.10, 14.90) | 14.10 (13.10, 15.00) | 14.10 (13.10, 14.80) | 0.6 |
| Lymphocyte number (1000 cells/uL) | 2.10 (1.70, 2.60) | 2.10 (1.70, 2.50) | 2.10 (1.60, 2.50) | 0.3 |
| Segmented neutrophils (1000 cell/uL) | 3.90 (3.00, 4.90) | 3.90 (3.00, 4.90) | 3.90 (2.90, 4.80) | 0.8 |
| Platelet count (1000 cells/uL) | 239 (204, 280) | 239 (203, 280) | 239 (201, 278) | 0.8 |
| Direct HDL-Cholesterol (mmol/L) | 1.32 (1.11, 1.58) | 1.32 (1.09, 1.60) | 1.32 (1.11, 1.60) | >0.9 |
| LDL-Cholesterol (mmol/L) | 2.74 (2.74, 2.74) | 2.74 (2.74, 2.74) | 2.74 (2.69, 2.74) | 0.7 |
| Triglyceride (mmol/L) | 1.01 (1.01, 1.01) | 1.01 (1.01, 1.01) | 1.01 (0.95, 1.01) | 0.4 |
| CPK (IU/L) | 116 (78, 173) | 116 (80, 177) | 116 (78, 176) | 0.5 |
| Total Cholesterol (mmol/L) | 4.73 (4.14, 5.38) | 4.73 (4.16, 5.43) | 4.73 (4.11, 5.35) | 0.7 |
| MASLD |  |  |  | >0.9 |
| No | 2,943 (62%) | 840 (62%) | 420 (62%) |  |
| Yes | 1,828 (38%) | 522 (38%) | 261 (38%) |  |
| *^1^* Median (Q1, Q3); n (%) | | | | |
| *^2^* Kruskal-Wallis rank sum test; Pearson’s Chi-squared test | | | | |

Abbreviations: ALP, alkaline phosphatase; ALT, alanine aminotransferase; AST, aspartate aminotransferase; CPK, Creatine Phosphokinase; GGT, gamma glutamyl transferase; HDL, HDL, high density lipoprotein; LDH, lactate dehydrogenase; LDL, low density lipoprotein; MASLD, metabolic dysfunction-associated steatotic liver disease; WBC, white blood cell count.
